# Editor's Letter-Box

**Published:** 1898-01-01

**Authors:** 


### Editor's Letter-Box.

[Our Correspondents are reminded that prolixity is a great bar to publication, and that brevity of style and conciseness of statement greatly facilitate early insertion.]

Mr. D. McLaren, of Hull, writes: In Hull we are in the midst of a rather heated discussion which will be of interest to you, and, if agreeable, I wish you would give the public the benefit of your opinion on the question. Several years ago (March, 1885) there was opened in Hull a hospital for the isolation of infectious cases by the Hull Corporation, and for a number of years no charge was made to any patient confined therein; but in the beginning of 1896 a resolution was passed by a majority to charge for all patients who the committee thought were in a position to pay. As you will see by the enclosed report, signed by the Chairman of the Sanitary Committee, this did not work satisfactorily, and in August, 1896, he gave notice of motion as on the same report. On October 6th, when the Council met, the following resolution was carried, "That the committee take no steps to recover the expenses incurred in maintaining patients in the general wards of the Infectious Diseases Hospital except in cases where, in the opinion of the committee, a patient can afford to pay for his maintenance in any such hospital." Carried by majority. During the twelve months about £37 has been paid, most persons refusing to pay, and no steps have been taken to recover. Last month an account of about £30 was pressed by Sanitary Committee and ordered to be sent to a Hull merchant for the maintenance of his children in hospital, and it gave rise to considerable discussion in the Council, but by a small majority it was agreed to ratify the committee's decision. At next Council meeting (December 9th) a resolution was moved to abandon all charges, and carried by 28 votes to 22. I enclose report of discussion. The next day the Sanitary Committee met, and gave notice to rescind the resolution at next meeting of the Council, January 6th, 1898. This was carried by 13 votes to 2, and two absent. The corporation hospitals have about 100 beds and a population of 225,000, although the Local Government Board recommend one bed to every 1,000 inhabitants. Huddersfield isolates 91 per cent. of its infectious cases, Hull 27 per cent. A considerable amount of hot blood is being created in this matter, and I ought to have added that there are five doctors on the Sanitary Committee, four of whom voted for charging both in committee and in council, and one (Dr. Briggs) against. I will just add that I am a member of the Hull Corporation, and hold the view that hospitals are built by authorities to prevent the spread of disease by isolation; consequently it is more those who are at liberty who should pay than those who are prisoners for the benefit of those free.

*** Notwithstanding section 132 of the Public Health Act, which allows the sanitary authority to recover from a patient, who is not a pauper, the cost of his maintenance, we think that the proper course is to open the wards of an isolation hospital freely to those suffering from the diseases it is intended to treat. Delay and difficulty in ensuring the removal of cases is sure to occur if a charge is made; and probably the worst possible arrangement is that which seems to have been in force lately at Hull, viz., making the charges become a debt, the payment of which has practically been optional. Such a plan, while interfering with the full use of the hospital almost as much as if payment were demanded, fails to get in the money, so that there is a loss at both ends. In our opinion an isolation hospital should be free, but should be well supplied with pay wards, which should be of a very superior character. When such wards are provided we may be pretty sure that well-to-do people, the people who really ought not to accept hospital treatment as a free gift from the municipality, will be very glad to pay, and even to pay handsomely, for the sake of having around them in sickness the privacy and the comforts of their own homes. The private wards at Croydon might be worth visiting.—Ed. *T.H.*

